# DURATION-2: efficacy and safety of switching from maximum daily sitagliptin or pioglitazone to once-weekly exenatide

**DOI:** 10.1111/j.1464-5491.2011.03301.x

**Published:** 2011-06

**Authors:** C Wysham, R Bergenstal, J Malloy, P Yan, B Walsh, J Malone, K Taylor

**Affiliations:** Rockwood ClinicSpokane, WA; * International Diabetes Center at Park NicolletMinneapolis, MN; † Amylin Pharmaceuticals Inc.San Diego, CA; ‡ Eli Lilly and CompanyIndianapolis, IN, USA

**Keywords:** exenatide, glucagon-like peptide 1, pioglitazone, sitagliptin, Type 2 diabetes

## Abstract

**Aims:**

In the initial 26-week, double-blind, double-dummy assessment period of the DURATION-2 trial in patients with Type 2 diabetes on metformin, the once-weekly glucagon-like peptide 1 (GLP-1) receptor agonist exenatide once-weekly resulted in greater HbA_1c_ improvement and weight reduction compared with maximum approved daily doses of sitagliptin or pioglitazone. This subsequent, 26-week, open-label, uncontrolled assessment period evaluated the safety and efficacy of (i) continued exenatide once-weekly treatment and (ii) switching from sitagliptin or pioglitazone to exenatide once-weekly.

**Methods:**

Randomised oral medications were discontinued and all patients received exenatide once-weekly. Of the 364 patients [original baseline HbA_1c_ 8.5 ± 1.1% (70 mmol/mol), fasting plasma glucose 9.0 ± 2.5 mmol/l, weight 88 ± 20 kg) who continued into the open-label period, 319 patients (88%) completed 52 weeks.

**Results:**

Evaluable patients who received only exenatide once-weekly demonstrated significant 52-week improvements (least square mean ± se) in HbA_1c_ (−1.6 ± 0.1%), fasting plasma glucose (−1.8 ± 0.3 mmol/l) and weight (−1.8 ± 0.5 kg). Evaluable patients who switched from sitagliptin to exenatide once-weekly demonstrated significant incremental improvements in HbA_1c_ (−0.3 ± 0.1%), fasting plasma glucose (−0.7 ± 0.2 mmol/l) and weight (−1.1 ± 0.3 kg). Patients who switched from pioglitazone to exenatide once-weekly maintained HbA_1c_ and fasting plasma glucose improvements (week 52: −1.6 ± 0.1%, −1.7 ± 0.3 mmol/l), with significant weight reduction (−3.0 ± 0.3 kg). Exenatide once-weekly was generally well tolerated and adverse events were predominantly mild or moderate in intensity. Nausea was the most frequent adverse event in this assessment period (intent-to-treat: exenatide once-weekly-only 5%; sitagliptin → exenatide once-weekly 11%; pioglitazone → exenatide once-weekly 10%). No major hypoglycaemia was observed.

**Conclusions:**

Patients who switched to once-weekly exenatide from daily sitagliptin or pioglitazone had improved or sustained glycaemic control, with weight loss.

## Introduction

In an attempt to address treatment concerns of many traditional medications for Type 2 diabetes, the most recent American Diabetes Association/European Association for the Study of Diabetes (ADA/EASD) [1] and American Association of Clinical Endocrinologists/American College of Endocrinology (AACE/ACE) [2] algorithms include, as adjunctive therapy to metformin, newer classes of medications that may positively influence some of the metabolic abnormalities associated with Type 2 diabetes (obesity, dyslipidaemia and other cardiovascular risk factors). In the DURATION-2 double-blind, double-dummy trial, three reasonable choices of add-on therapy were directly compared in patients with Type 2 diabetes not adequately controlled with metformin monotherapy. After 26 weeks of treatment, once-weekly dosing with the glucagon-like peptide 1 (GLP-1) receptor agonist exenatide resulted in superior improvements in glycaemic control and body weight compared with maximum approved doses of the dipeptidyl peptidase-4 (DPP-4) inhibitor, sitagliptin, and the thiazolidinedione, pioglitazone [3].

In the subsequent, open-label phase of the DURATION-2 trial, all patients received exenatide once-weekly. Rather than adding another medication, as is common in clinical practice, patients who originally received daily sitagliptin or pioglitazone discontinued their respective oral treatment to assess incremental changes in glycaemic control and body weight after switching to once-weekly administration of exenatide. Patients originally randomised to exenatide once-weekly continued treatment and discontinued oral placebo. In this open-label extension period, all patients continued metformin background therapy, and patients and investigators remained blinded to original treatment. This trial was designed to evaluate the glycaemic and non-glycaemic (body weight, blood pressure, fasting lipids, markers of cardiovascular risk) changes in patients: (i) treated with exenatide once-weekly for 52 weeks; (ii) who switched from daily inhibition of DPP-4 (which elicits an approximately twofold increase in circulating postprandial GLP-1 [4]) to a therapy that results in continuous exposure to greater concentrations of the GLP-1 receptor agonist exenatide; and (iii) who switched from daily peroxisome proliferator-activated receptor gamma (PPAR-γ) stimulation with pioglitazone, which increases insulin sensitivity, but is also often associated with increased body weight, to a therapy that augments glucose-dependent insulin secretion and is associated with weight loss.

## Patients and methods

### Design overview

DURATION-2, a randomised, double-blind, double-dummy, multi-center clinical trial conducted in the USA, India and Mexico was designed as a two-stage trial. In the first phase, the efficacy and safety of 26 weeks of treatment with exenatide once-weekly were compared with maximum approved doses of sitagliptin or pioglitazone in patients with Type 2 diabetes mellitus treated with metformin [3]. In this second, open-label phase, randomised oral medications were discontinued and all patients received exenatide once-weekly.

### Randomization and interventions

Eligible patients were male and non-pregnant female patients, at least 18 years of age, with Type 2 diabetes mellitus, but otherwise healthy and treated with a stable regimen of metformin for a minimum of 2 months prior to screening. Additional inclusion criteria included HbA_1c_ 7.1–11.0% (54–97 mmol/mol) and BMI 25–45 kg/m^2^ [3]. Patients (*n* = 514) were originally randomised to one of three treatment groups by UBC Clinical Technologies (San Francisco, CA, USA) via an interactive voice response system: exenatide once-weekly (2 mg) subcutaneous injection (self-administered) plus placebo once daily in the morning capsule, sitagliptin (100 mg) once daily in the morning plus placebo once-weekly injection, or pioglitazone (45 mg) once daily in the morning plus placebo once-weekly injection. All patients, the study-site staff, the investigator and the sponsor were blinded to the identity of study medication during the double-blind treatment period. Randomization was stratified according to country and screening HbA_1c_ stratum [< 9% (< 75 mmol/mol), ≥ 9% (≥ 75 mmol/mol)]. In the second, open-label phase of DURATION-2, randomised oral medications were discontinued and all patients received 2 mg exenatide once-weekly treatment; patients and investigators were not notified of original treatment. A common clinical protocol was approved for each site by the appropriate Ethical Review Board. Patients provided written informed consent prior to participation. This study was conducted in accordance with the Declaration of Helsinki (1946), including the current Seoul revision (2008), and consistent with Good Clinical Practice and applicable laws and regulations.

### Outcomes

Study endpoints included change from baseline to weeks 26 and 52, and change from week 26 to week 52, in HbA_1c_, patients achieving HbA_1c_ targets of < 7.0% (< 53 mmol/mol) and ≤ 6.5% (≤ 48 mmol/mol), change in fasting plasma glucose (target ≤ 7 mmol/l), body weight, fasting lipids, fasting insulin, systolic and diastolic blood pressure, cardiovascular risk markers (urinary albumin/creatinine ratio, B-type natriuretic peptide, high-sensitivity C-reactive protein and plasminogen activator inhibitor-1), safety and tolerability. Plasma and urine analytes and HbA_1c_ were quantitated by Quintiles Laboratories (Marietta, GA, USA; Mumbai, India; and Singapore) using standard methods. All other measures were performed as previously described [3].

Treatment-emergent adverse events were defined as those occurring for the first time or worsening after the first dose of exenatide once-weekly during the open-label period. Hypoglycaemia was categorized as major or minor. Major hypoglycaemia was defined as being associated with (i) a loss of consciousness, seizure or coma, which resolved after administration of glucagon or glucose or (ii) severe impairment in consciousness or behaviour that required third-party assistance to resolve and a glucose value of < 3 mmol/l. Minor hypoglycaemia was defined as a report of symptoms consistent with hypoglycaemia and a glucose value of < 3 mmol/l prior to treatment of the episode.

### Statistical analysis

**Figure 1:**
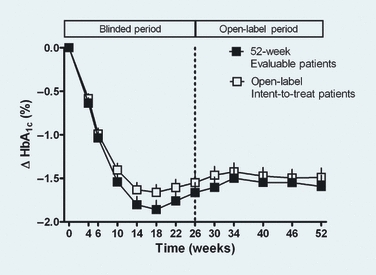
Least square mean ± se HbA_1c_ change over 52 weeks in 52-week evaluable and open-label intent-to-treat (ITT) patients who were originally randomised to receive exenatide once weekly at the start of the study.

The open-label intent-to-treat population used for all safety analyses consisted of all subjects who completed the 26-week double-blinded treatment period and they received one dose of open-label once-weekly exenatide at a timepoint after week 26. The 52-week evaluable population consisted of the open-label intent-to-treat patients who completed the study visits to at least week 46 in compliance with the protocol and received adequate study medication exposure in this assessment period (≥ 145 days of exenatide once-weekly; ≤ 2 doses missed during the last 2 months). There were no substantial differences in HbA_1c_ between the exenatide once-weekly-only 52-week evaluable and intent-to-treat populations (Fig. 1); therefore, demographics and the analysis of non-safety endpoints are provided for the 52-week evaluable population.

Analyses of change in HbA_1c_ from baseline to weeks 26 and 52 were based on a general linear model, including factors for treatment, country and baseline HbA_1c_ stratum. Change in HbA_1c_ during the open-label phase (weeks 26–52) was analysed based on a general linear model, including factors for treatment, country and HbA_1c_ values at week 26 as a covariate. Analyses of change in other variables from baseline to weeks 26 and 52 were based on a general linear model, including factors for treatment, country, baseline HbA_1c_ stratum and corresponding baseline value as a covariate. Change in other variables during the open-label phase were analysed based on a general linear model, including factors for treatment, country and corresponding values of variables at week 26 as a covariate. Log-transformation was applied to triglycerides and cardiovascular risk markers before fitting the model. Comparison of proportions achieving HbA_1c_ and fasting plasma glucose targets between week 26 and week 52 within each treatment group was performed using a McNemar test. Mean change from baseline for efficacy endpoints was expressed as least square means. Statistical tests were conducted two-sided at a significance level of 0.05 using SAS (version 9.2; SAS Institute, Cary, NC, USA).

## Results

### Patient disposition and baseline characteristics

**Figure 2:**
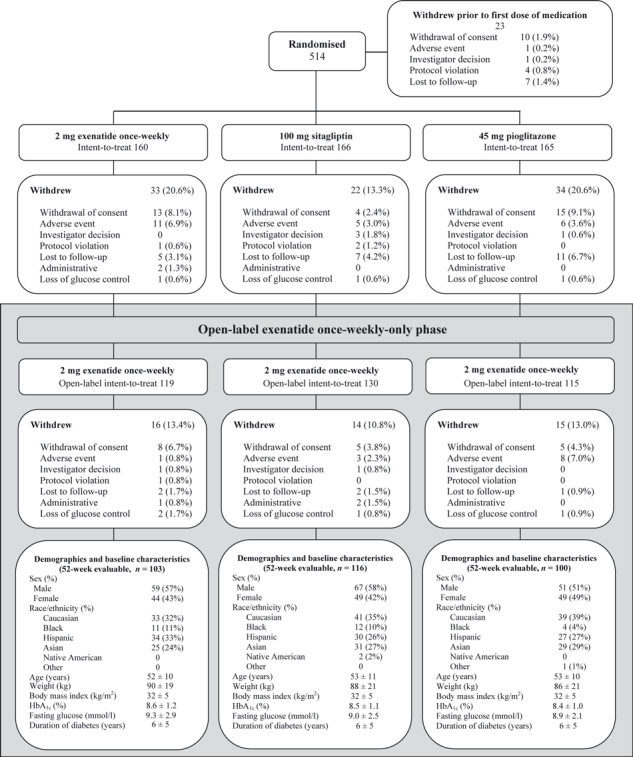
Patient disposition and demographics [mean ± sd or *n* (%)].

Demographics and baseline characteristics for the 52-week evaluable population were similar between treatment arms (Fig. 2) and 87–89% of patients completed this 26-week open-label period, regardless of initial therapy. The most common reason for withdrawal was withdrawal of consent in patients originally receiving exenatide once-weekly (7%) and sitagliptin (4%), while withdrawal because of an adverse event was most common (7%) among patients originally randomised to pioglitazone.

### Glycaemic control

**Figure 3:**
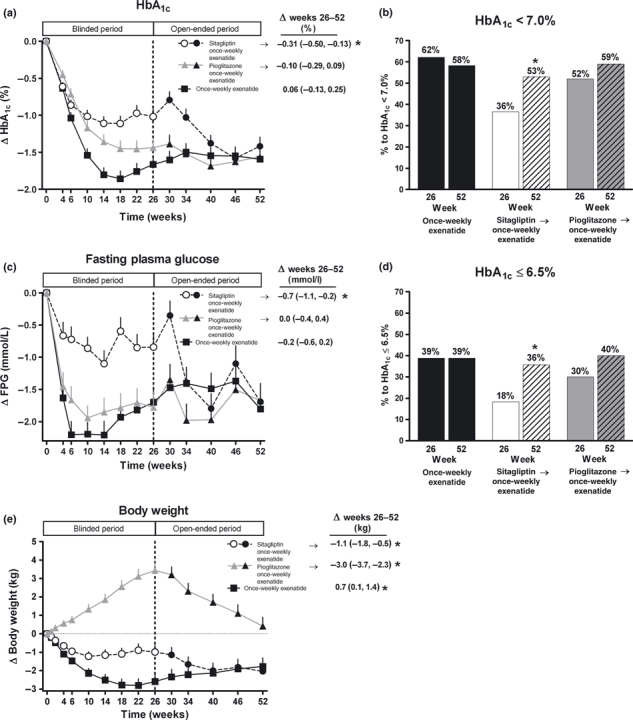
Change in glycaemic control and body weight. (a) Least square (LS) mean ± se change in HbA_1c_ in 52-week evaluable patients over 52 weeks. Change from week 26 to week 52 is presented in figure insert. (b) Per cent of 52-week evaluable patients achieving HbA_1c_ target < 7% (< 53 mmol/mol) at week 26 and week 52. (c) LS mean ± se change in fasting plasma glucose in 52-week evaluable patients over 52 weeks. Change from week 26 to week 52 is presented in figure insert. (d) Per cent of 52-week evaluable patients achieving HbA_1c_ target ≤ 6.5% (≤ 48 mmol/mol) at week 26 and week 52. (e) LS mean ± se change in body weight in 52-week evaluable patients over 52 weeks. Change from week 26 to week 52 is presented in figure insert. *P < 0.05.

Patients who received exenatide once-weekly for 52 weeks sustained the initial 26-week improvements in HbA_1c_ [least square mean change from original baseline (95% CI)] at week 52: −1.6% (−1.9 to −1.3%); Fig. 3. Patients who switched from pioglitazone to exenatide once-weekly treatment also maintained HbA_1c_ improvement [week 52: −1.6% (95% CI −1.8 to −1.3%)], while patients who switched from sitagliptin to exenatide once-weekly exhibited a significant incremental reduction in HbA_1c_ (−0.3%; *P* = 0.0010), resulting in a 52-week HbA_1c_ reduction of −1.4% (95% CI −1.7 to −1.2%). No significant changes in the proportion of patients who achieved the HbA_1c_ targets of < 7% (< 53 mmol/mol) and ≤ 6.5% (≤ 48 mmol/mol) at week 52 compared with week 26 were observed for patients who originally received exenatide once-weekly or pioglitazone; significantly more patients achieved both HbA_1c_ targets after switching from sitagliptin to exenatide once-weekly (Fig. 3).

Exenatide once-weekly-only and pioglitazone → exenatide once-weekly patients also maintained fasting plasma glucose improvements from week 26 to week 52 (Fig. 3), while patients originally randomised to sitagliptin exhibited significant further fasting plasma glucose improvements (−0.7 mmol/l; *P* = 0.0017). At the end of this assessment period, the proportion of patients who achieved fasting plasma glucose ≤ 7 mmol/l was unchanged with exenatide once-weekly-only (week 26 → week 52: 62% → 62%) and following the switch from pioglitazone to exenatide once-weekly (week 26 → week 52: 59% → 59%). Significantly more patients achieved fasting plasma glucose ≤ 7 mmol/l after switching from sitagliptin to exenatide once-weekly (week 26 → week 52: 38% → 58%; *P* = 0.0002).

### Body weight

Patients who switched from pioglitazone to exenatide once-weekly exhibited a significant weight reduction (−3.0 kg; *P* < 0.0001; Fig. 3), such that body weight was similar to original baseline weight by week 52. Patients originally treated with sitagliptin also exhibited a significant reduction in weight after switching to exenatide once-weekly (−1.1 kg; *P* = 0.0006). Patients who received exenatide once-weekly throughout the trial on average had a + 0.7 kg (*P* = 0.0325) increase in weight from week 26 to week 52, resulting in a −1.8 ± 0.5 kg (*P* = 0.0002) reduction from original baseline.

### Markers of cardiovascular risk

Systolic blood pressure changes are presented in Table 1. Patients with abnormal baseline systolic blood pressure (≥ 130 mmHg) treated with exenatide once-weekly for 52 weeks (*n* = 42) exhibited greater systolic blood pressure improvements [−12.2 mmHg (95% CI −16.1 to −8.3)]. Patients who exhibited abnormal systolic blood pressure at week 26 after treatment with sitagliptin (*n* = 46) or pioglitazone (*n* = 34) also demonstrated a greater systolic blood pressure reduction [−11.3 mmHg (95% CI −14.9 to −7.7) and −9.4 mmHg (95% CI −13.4 to −5.3), respectively; week 26 to week 52]. Changes in concomitant anti-hypertensive medications were only allowed if deemed necessary by the investigator. Of the 186 patients who used an agent to treat hypertension at the start of the open-label assessment period, 172 patients (99, 87 and 93% of patients originally randomised to exenatide once-weekly, sitagliptin and pioglitazone, respectively) did not change dose and two patients (*n* = 1 sitagliptin → exenatide once-weekly; *n* = 1 pioglitazone → exenatide once-weekly) initiated medication after the start of the open-label period.

**Table 1:** Blood pressure and fasting lipids (52-week evaluable population)

	Exenatide once-weekly → exenatide once-weekly *n* = 103	Sitagliptin → exenatide once-weekly *n*= 116	Pioglitazone → exenatide once-weekly *n* = 100
Blood pressure (mmHg)			
SBP (baseline)	127 ± 1	126 ± 1	126 ± 1
Δ weeks 26–52	−0.9 (−3.2 to 1.5)	−2.7 (−4.9 to −0.5)*	−0.6 (−3.0 to 1.7)
Δ weeks 0–52	−2.9 (−5.4 to −0.4) *	−2.9 (−5.3 to −0.6) *	−2.2 (−4.7 to 0.3)
DBP (baseline)	80 ± 1	78 ± 1	79 ± 1
Δ weeks 26–52	−1.2 (−2.7 to 0.3)	−0.4 (−1.9 to 1.0)	+1.1 (−0.5 to 2.6)
Δ weeks 0–52	−2.1 (−3.7 to −0.6) *	−1.3 (−2.8 to 0.2)	−0.4 (−2.0 to 1.1)
Blood lipids (mmol/l)			
Total cholesterol (baseline)	4.61 ± 0.11	4.59 ± 0.10	4.79 ± 0.11
Δ weeks 26–52	−0.07 (−0.21 to 0.07)	−0.26 (−0.40 to −0.12)*	−0.34 (−0.48 to −0.19)*
Δ weeks 0–52	−0.05 (−0.20 to 0.10)	−0.20 (−0.34 to −0.06)*	−0.22 (−0.37 to −0.07) *
LDL (baseline)	2.77 ± 0.09	2.74 ± 0.08	2.91 ± 0.10
Δ weeks 26–52	0.00 (−0.12 to 0.13)	−0.09 (−0.20 to 0.03)	−0.14 (−0.27 to −0.02)*
Δ weeks 0–52	−0.03 (−0.17 to 0.10)	−0.09 (−0.21 to 0.04)	−0.12 (−0.25 to 0.02)
HDL (baseline)	1.06 ± 0.02	1.10 ± 0.02	1.08 ± 0.03
Δ weeks 26–52	0.00 (−0.03 to 0.03)	−0.01 (−0.04 to 0.02)	−0.14 (−0.18 to −0.11)*
Δ weeks 0–52	0.07 (0.04 to 0.11) *	0.06 (0.02 to 0.09) *	0.01 (−0.03 to 0.05)
Triglycerides (baseline)†	1.80 ± 0.09	1.63 ± 0.07	1.93 ± 0.09
Δ weeks 26–52	10% (2 to 19) *	2% (−5 to 9)	11% (3 to 20)*
Δ weeks 0–52	0% (−7 to 8)	−6% (−12 to 1)	−7% (−14 to 1)

Baseline data are mean ± se for total cholesterol, LDL and HDL.

Least square mean (95% CI) for change from baseline or week 26 for total cholesterol, LDL and HDL.

† Geometric mean ± se for triglycerides baseline, and geometric least square mean % change (95% CI) for change from baseline to week 26 for triglycerides.

Δ Change from previous value

* *P* < 0.05.

DBP, diastolic blood pressure; SBP, systolic blood pressure.

Patients who continued exenatide once-weekly for 52 weeks maintained improvement in HDL cholesterol from baseline; all other lipid variables were not significantly changed from original baseline (Table 1). Patients who switched from sitagliptin to exenatide once-weekly also maintained the HDL cholesterol improvement observed during the initial 26 weeks of treatment and experienced a significant reduction in total cholesterol in this open-label treatment period. Patients who switched from pioglitazone to exenatide once-weekly significantly reduced HDL cholesterol, LDL cholesterol and total cholesterol and increased triglycerides. Of the 132 patients who used a lipid-lowering agent at the start of the 26-week open-label assessment period, 121 patients (91, 89 and 95% of patients originally randomised to exenatide once-weekly, sitagliptin and pioglitazone, respectively) did not change dose and 13 patients (*n* = 3 exenatide once-weekly-only; *n* = 5 sitagliptin → exenatide once-weekly; *n* = 5 pioglitazone → exenatide once-weekly) started a lipid-lowering medication after the start of the open-label period.

Exenatide once-weekly for 52 weeks was associated with improvements in the urinary albumin/creatinine ratio, B-type natriuretic peptide and high-sensitivity C-reactive protein (Table 2). Although the mean urinary albumin/creatinine ratio reductions in both groups that switched to exenatide once-weekly were not statistically significant during this treatment period, the urinary albumin/creatinine ratio was significantly reduced from original baseline in all three treatment arms. Of note, urinary creatinine was not significantly different from baseline to week 52 in any treatment arm. Patients who switched from sitagliptin or pioglitazone to once-weekly exenatide significantly reduced B-type natriuretic peptide, while the high-sensitivity C-reactive protein and plasminogen activator inhibitor-1 improvements observed after 26 weeks of pioglitazone treatment were not maintained with exenatide once-weekly treatment.

**Table 2:** Markers of cardiovascular risk (52-week evaluable population)

	Markers of cardiovascular risk		
	
	Exenatide once-weekly → exenatide once-weekly *n* = 103	Sitagliptin → exenatide once-weekly *n* = 116	Pioglitazone → exenatide once-weekly *n* = 100
ACR (baseline)	15.31 ± 2.37	11.46 ± 1.27	13.23 ± 1.92
Δ weeks 26–52	−19% (−31 to −5)*	−14% (−26 to 0)	−12% (−25 to 4)
Δ weeks 0–52	−34% (−45 to −20)*	−18% (−31 to −3)*	−23% (−36 to −7)*
BNP (baseline; pg/ml)	9.66 ± 1.00	11.69 ± 1.03	9.60 ± 0.84
Δ weeks 26–52	−10% (−23 to 6)	−16% (−27 to −3)*	−26% (−37 to −14)*
Δ weeks 0–52	−18% (−31 to −3)*	−15% (−28 to −1)*	−13% (−26 to 3)
hsCRP (baseline; mg/l)	2.50 ± 0.24	2.35 ± 0.18	2.33 ± 0.24
Δ weeks 26–52	−2% (−15 to 12)	−8% (−20 to 5)	37% (19 to 58)*
Δ weeks 0–52	−25% (−35 to −13)*	−17% (−27 to −4)*	−5% (−18 to 11)
PAI-1 (baseline; ng/ml)	39.14 ± 2.29	32.97 ± 1.71	36.18 ± 1.95
Δ weeks 26–52	16% (4 to 30)*	−3% (−12 to 8)	27% (14 to 42)*
Δ weeks 0–52	4% (−8 to 16)	−8% (−18 to 2)	12% (0 to 25)

Baseline data are geometric mean ± se. Geometric least square mean % change (95% CI) for change from baseline or week 26.

* *P* < 0.05.

Δ Change from previous value

ACR, urinary albumin/creatinine ratio; BNP, B-type natriuretic peptide; hsCRP, high-sensitivity C-reactive protein; PAI-1, plasminogen activator inhibitor 1.

### Safety and tolerability

The most frequent treatment-emergent adverse events in this assessment period were nausea and diarrhoea (Table 3). Nine patients experienced 13 treatment-emergent serious adverse events. The only serious adverse event that occurred in more than one patient was anxiety (*n* = 2), but did not lead to withdrawal and was deemed unrelated to study medication. Of the 45 patients who withdrew during this study period, the majority (64%) withdrew because of withdrawal of consent, investigator decision, protocol violation, lost to follow-up or for administrative reasons (Fig. 2). Sixteen patients withdrew because of either loss of glucose control (*n* = 4) or an adverse event (*n* = 12) during this treatment period. The only treatment-emergent adverse events leading to withdrawal in more than one patient were abdominal pain and nausea (Table 3). No cases of pancreatitis were reported in this 26-week open-label treatment period and no cases were reported in patients receiving exenatide once-weekly during the first 26 weeks of the study [3]. No episodes of major hypoglycaemia occurred; minor hypoglycaemia incidence was low [*n* = 5 (1%)] and was similar among cohorts (exenatide once-weekly-only: 1%; sitagliptin → exenatide once-weekly: 2%; pioglitazone → exenatide once-weekly: 1%).

**Table 3:** Frequent adverse events (≥ 5% in any cohort) and adverse events leading to withdrawal during the open-ended extension period (weeks 26 to 52; open-label intent-to-treat population)

	Exenatide once-weekly → exenatide once-weekly *n* = 119	Sitagliptin → exenatide once-weekly *n* = 130	Pioglitazone → exenatide once-weekly *n* = 115
Frequent adverse events (≥ 5%)†			
Diarrhoea	10 (8.4) 12	10 (7.7) 13	9 (7.8) 12
Upper respiratory tract infection	7 (5.9) 7	6 (4.6) 7	7 (6.1) 7
Nausea	6 (5.0) 6	14 (10.8) 21	11 (9.6) 13
Vomiting	6 (5.0) 6	5 (3.8) 5	3 (2.6) 4
Nasopharyngitis	6 (5.0) 6	3 (2.3) 3	5 (4.3) 6
Urinary tract infection	4 (3.4) 4	6 (4.6) 10	7 (6.1) 10
Dyspepsia	0	2 (1.5) 2	7 (6.1) 7
Treatment-emergent adverse events leading to withdrawal‡			
Total	2 (1.7)	4 (3.1)	8 (7.0)
Abdominal distension	0	0	1 (0.9)
Abdominal pain	0	0	2 (1.7)
Alanine aminotransferase increased	1 (0.8)	0	0
Blood creatine phosphokinase increased	1 (0.8)	0	0
Cholelithiasis	0	0	1 (0.9)
Decreased appetite	0	1 (0.8)	0
Hepatitus B surface antigen positive	0	0	1 (0.9)
Lipase increased	0	0	1 (0.9)
Nausea	0	2 (1.5)	2 (1.7)
Rash	0	1 (0.8)	0

* Adverse events occurred for the first time or worsened after the first dose of exenatide once-weekly during the open-label period.

† *n* (%) events for frequent adverse events.

‡ *n* (%) for individual treatment-emergent adverse events leading to withdrawal (withdrawal may have occurred after week 52).

## Discussion

### Glycaemic control and body weight

Exenatide once-weekly elicits continuous exposure to therapeutic concentrations of the GLP-1 receptor agonist, exenatide, which acts to increase glucose-dependent insulin section, decrease inappropriate elevations in postprandial glucagon, increase satiety and slow gastric emptying [5]. The continuous exenatide exposure achieved with exenatide once-weekly has been shown to result in a significantly greater fasting plasma glucose and HbA_1c_ reduction, with similar weight reduction, compared with the twice daily exenatide formulation [6]. In the current study, patients who continued exenatide once-weekly treatment for a total of 52 weeks maintained the improvements in HbA_1c_ and fasting plasma glucose observed during the first 26 weeks of treatment (52-week improvements: −1.6% and −1.8 mmol/l) and exhibited a significant improvement from original baseline in body weight (−1.8 kg). Similar 52-week improvements in HbA_1c_, fasting plasma glucose and weight have previously been demonstrated [7].

DPP-4 inhibitors (e.g. sitagliptin) and GLP-1 receptor agonists (e.g. exenatide) elicit effects through the GLP-1 pathway; however, distinct differences exist in the mechanism of action of these compounds. Namely, inhibition of the DPP-4 leads to a two- to threefold elevation of endogenous GLP-1, while administration of GLP-1 receptor agonists results in higher plasma concentrations of agents that bind directly to the GLP-1 receptor. In a short-term trial by DeFronzo *et al*. comparing exenatide twice daily with sitagliptin [4], the postprandial circulating concentration of exenatide, which has similar potency to GLP-1 [8,9], was approximately fourfold greater than the increase in postprandial GLP-1 achieved with sitagliptin (15 pm; [4]). The greater circulating GLP-1 receptor agonist concentration observed with exenatide twice daily in the study by DeFronzo et al. [4] was associated with greater postprandial glucose control and body weight reduction, and similar fasting plasma glucose improvement, compared with sitagliptin. The exenatide molecule in the sustained release (once-weekly) formulation used in the present study and twice daily formulation are identical, and the primary mechanism for the enhanced efficacy of exenatide once-weekly compared with sitagliptin during the first 26 weeks of this study [3] was likely due to the continuous exposure to therapeutic concentrations of exenatide (∼70 pm; [6]) achieved with once-weekly exenatide. Thus, the significant improvement in HbA_1c_, fasting plasma glucose and body weight observed in the present study after switching from sitagliptin to exenatide once-weekly may be attributable to the continuous exposure to higher circulating concentrations of the GLP-1 receptor agonist exenatide.

Pioglitazone, which exerts its anti-hyperglycaemic effects primarily through decreasing insulin resistance, resulted in a significantly smaller HbA_1c_ improvement and similar fasting plasma glucose improvement compared with patients treated with exenatide once-weekly in the first phase of this trial [3]. In the cohort of these patients who completed 52 weeks of treatment, exenatide once-weekly maintained HbA_1c_ and fasting plasma glucose improvements; however, weight significantly decreased to a value similar to that observed prior to pioglitazone treatment.

### Markers of cardiovascular risk

Consistent with a previous study of exenatide once-weekly [7], patients who received exenatide once-weekly for 52 weeks maintained significant systolic blood pressure improvement (∼3 mmHg). Systolic blood pressure was not significantly changed in patients originally randomised to sitagliptin during the initial 26-week period [3]; however, switching to exenatide once-weekly was associated with significantly reduced systolic blood pressure. Of note, the improvement in systolic blood pressure was greater in patients with abnormal (≥ 130 mmHg) systolic blood pressure.

In contrast to a previous assessment of exenatide once-weekly [7], a significant 52-week improvement in HDL cholesterol, but not total cholesterol and triglycerides, was observed. The reasons for this discrepancy are unclear, as the baseline values for these lipid variables were comparable. The first phase of this study [3] confirmed the well-established effects of pioglitazone [10] to decrease triglycerides and increase total and HDL cholesterol. Treatment with exenatide once-weekly in these patients increased triglycerides and decreased LDL and HDL, such that these lipids were not significantly changed from original baseline. While a significant change in total cholesterol was not observed in patients treated with exenatide once-weekly for 52 weeks, switching to exenatide once-weekly from either sitagliptin or pioglitazone was associated with a significant improvement in total cholesterol.

Overall, treatment with exenatide once-weekly for 52 weeks, or switching to exenatide once-weekly from sitagliptin, was associated with favourable 52-week changes in the urinary albumin/creatinine ratio, B-type natriuretic peptide and high-sensitivity C-reactive protein. Patients who switched from pioglitazone exhibited a significant decrease in B-type natriuretic peptide, and increases in high-sensitivity C-reactive protein and plasminogen activator inhibitor-1, such that these markers were not significantly changed from original baseline; the urinary albumin/creatinine ratio significantly improved from original baseline. It should be noted that the low mean baseline values of these markers make clinical interpretations difficult. Although the mechanism responsible for these improvements cannot be elucidated from this study, it has been demonstrated that improvements in microalbuminuria are associated with improvements in systolic blood pressure and HbA_1c_ [11]; B-type natriuretic peptide is also interrelated with renal and vascular function [12]. The differential effects by which exenatide increased high-sensitivity C-reactive protein to original baseline values in pioglitazone-treated patients, but was associated with overall high-sensitivity C-reactive protein improvements in the other two cohorts are unclear but may be related to the similar differences in HDL cholesterol change between these treatment arms (HDL cholesterol has been suggested to be the primary lipid associated with high-sensitivity C-reactive protein elevation [13]; Table 1). Further studies are warranted to elucidate the effects of exenatide once-weekly on these cardiovascular risk markers, particularly in patients with abnormal values.

### Safety and tolerability

As in previous trials with exenatide, mild to moderate gastrointestinal events were the primary adverse events. Of note, patients who received exenatide once-weekly during the initial 26-week treatment period experienced a lower rate of nausea compared with patients who switched (5 vs. 10–11%), presumably because of prior exenatide exposure. Consistent with the glucose-dependent nature of enhanced insulin secretion with exenatide, there were no episodes of major hypoglycaemia throughout the study. Overall, 16 patients (4%) withdrew during this period as a result of an adverse event or loss of glucose control.

## Conclusion

The open-label, uncontrolled, study design limits the conclusions that can be drawn from this study period; however, it should be noted that, in this phase of the study, neither the patients nor the investigators were informed of original treatment assignment (exenatide once-weekly, sitagliptin or pioglitazone) during the initial 26-week treatment period. The 52-week improvements in glycaemic control and body weight associated with exenatide once-weekly-only treatment were similar to those previously reported [7]. The improvements in glycaemic control, body weight and systolic blood pressure observed following the switch from sitagliptin to exenatide once-weekly were consistent with continuous exposure to higher pharmacological concentrations of a GLP-1 receptor agonist. Furthermore, the maintenance of glycaemic control and accompanying weight reduction observed following the switch from pioglitazone to exenatide once-weekly may be of particular clinical significance for some patients. Of note, exenatide once-weekly has also demonstrated greater improvement in HbA_1c_ and body weight compared with titrated insulin glargine, with a lower incidence of hypoglycaemia but with a greater incidence of gastrointestinal adverse events [14]. Thus, exenatide once-weekly has been demonstrated to elicit greater HbA_1c_ improvement when directly compared with three agents that are commonly used after metformin (sitagliptin, pioglitazone, insulin glargine). The current findings suggest that exenatide once-weekly may also be a suitable treatment alternative in patients with Type 2 diabetes on a background of metformin who are not currently achieving adequate glycaemic control with a DPP-4 inhibitor, or in whom thiazolidinedione-related body weight gain is a concern.

## Abbreviations

DPP-4: dipeptidyl peptidase-4
GLP-1: glucagon-like peptide 1
